# Evaluating a web-based computer-tailored physical activity intervention for those living with and beyond lung cancer (ExerciseGuide UK): protocol for a single group feasibility and acceptability study

**DOI:** 10.1186/s40814-022-01129-6

**Published:** 2022-08-13

**Authors:** Jordan Curry, Michael Lind, Camille E. Short, Corneel Vandelanotte, Holly E. L. Evans, Mark Pearson, Cynthia C. Forbes

**Affiliations:** 1 Wolfson Palliative Care Research Centre, Hull York Medical School, University of Hull, Allam Medical Building 3rd Floor, Cottingham Road, Kingston-Upon-Hull, East Yorkshire HU6 7RX UK; 2 Academic Department of Oncology, Queen’s Centre for Oncology and Haematology, Castle Hill Hospital, Cottingham, Hull, UK; 3 Melbourne Centre for Behaviour Change, Faculty of Medicine, Dentistry, and Health Sciences, The University of Melbourne, Parkville, Victoria Australia; 4 Appleton Institute, Physical Activity Research Group, Central Queensland University, North Rockhampton, Queensland Australia; 5 Freemasons Foundation Centre for Men’s Health, School of Medicine, University of Adelaide, Adelaide, South Australia Australia; 6 iNform Research Institute, iNform Health and Fitness, Adelaide, South Australia Australia

**Keywords:** Lung cancer, Physical activity, Exercise, Telehealth, eHealth, Feasibility, Acceptability, User-friendliness, Usability

## Abstract

**Background:**

Lung cancer is the leading cause of cancer-related death globally. Physical activity and exercise provide unequivocal benefits to those living with and beyond lung cancer. However, few of those living with and beyond cancer meet the national physical activity guidelines. Various barriers exist for this population’s engagement in physical activity and exercise, such as the lack of knowledge and lack of tailored information, little access to exercise specialists, fatigue, and mobility challenges. Digitally delivered programmes have the potential to address several of these barriers, with techniques like “computer-tailoring” available to enable the delivery of tailored content at a time and place that is convenient. However, evaluation of such programmes is needed prior to implementation. This protocol describes a single group study that will examine the feasibility and acceptability of an online tool (ExerciseGuide UK) that provides those living with and beyond lung cancer web-based computer-tailored physical activity prescription and modules underpinned by behaviour change theories.

**Methods:**

Thirty-five individuals diagnosed with lung cancer, or cancer affecting the lung (e.g. pleural mesothelioma), will be recruited into a single-intervention arm. The platform will provide tailored resources and a personalised physical activity programme using IF-THEN algorithms. Exercise prescription will be tailored on factors such as self-reported specific pain location, exercise history, and current physical fitness. In addition, modules grounded in behaviour change will supplement the physical activity programme and will focus on topics such as exercise benefits, safety, goal setting, and tracking. The primary outcome will be assessed using pre-established criteria on feasibility and mixed-methods approach for acceptability.

Secondary outcomes will explore changes in the physical activity, quality of life, anxiety, and depression.

**Discussion:**

This manuscript describes the protocol for a study examining the feasibility and acceptability of a web-based computer-tailored physical activity intervention for those living with and beyond lung cancer. The publication of this protocol aims to increase the transparency of the methods, report pre-determined criteria, and aid replication of the study and associated materials. If feasible and acceptable, this intervention will inform future studies of digital-based interventions.

**Trail registration:**

ClinicalTrails.gov, NCT05121259. Registered on November 16, 2021.

**Supplementary Information:**

The online version contains supplementary material available at 10.1186/s40814-022-01129-6.

## Introduction

Lung cancer is the second most diagnosed malignancy and the leading cause of cancer-related death globally [1]. Those living with and beyond lung cancer (LWBLC) may experience several curative treatment procedures, including chemotherapy, radiotherapy, immunotherapy, and surgery. Though potentially lifesaving, these treatments can lead to and exacerbate a number of long-lasting symptoms such as fatigue, loss of cardiorespiratory fitness, pain, and breathlessness [2–6]. Often, those LWBLC report feelings of low mood and depression, as they are forced [2–4] to accept the changes to their life caused by a diagnosis of cancer [7].

Physical activity and exercise are often used interchangeably within literature, though they are not synonymous [8]. Physical activity is defined as any bodily movement caused by the skeletal muscle which results in energy expenditure [9, 10]. Exercise is a subset of physical wherein an individual is in physical activity in a purposive, structured, and repetitive manner, with the intention of improving or maintaining one or more components of physical fitness [9, 11]. Engaging in a regular physical activity and exercise, in particular, is a recommended strategy for improving health and quality of life among cancer patients [12]. The American College of Sports Medicine (ACSM) has highlighted the benefits of physical activity that can elicit for those living with and beyond cancer [13]. Despite the increase in research in this area, the most robust evidence base is still primarily drawn from early-stage breast and prostate cancer survivors.

Nevertheless, amongst those LWBLC, physical activity and exercise has demonstrated to have several positive biological and physiological effects, such as reducing fatigue, anxiety, and depression while increasing muscle strength, increasing quality of life, and mitigating treatment side effects [14]. Furthermore, evidence supports the guidance for those living with and beyond cancer, including those LWBLC, to increase their physical activity post-diagnosis to increase survival outcomes [15, 16]. Cancer cachexia is a multifaceted syndrome with progressive loss of the skeletal muscle [17] and occurs in 20% of early stage lung cancer [18], 40% metastatic non-small cell lung cancer [19], and up to 69% of advanced lung cancer [20]. Increasing muscle strength and muscle mass may be beneficial in reducing the rate of muscular wasting and cancer cachexia [21]. However, the majority of this population worldwide does not meet the physical activity guidelines [22–25]. There are a multitude of reasons for this. In addition to common barriers to physical activity such as lack of time, access to facilities, and motivation, people LWBLC have disease-specific barriers. These can include fear of breathlessness, fatigue, pain, lack of knowledge about activity, symptom burdens, mood, and fear [14, 26]. People LWBLC have also reported a lack of physical activity recommendations and advice from oncology clinicians [27]. There is a clear need to explore and develop new supportive and survivorship care methods to better support patients. A study by Lin and colleagues (2013) interviewed people LWBLC and found that 70.4% of patients showed an interest in physical activity programmes. Furthermore, 69.1% of patients LWBLC reported they had the ability to participate in physical activity programmes [28].

A meta-analysis of patient-level data reported that supervised exercise programmes yield a greater quality of life and physical functioning improvements than unsupervised programmes [29], though both supervised and unsupervised exercise programmes were better than usual care control groups. Supervised programmes are thought to have greater efficacy owing in part to greater ability to provide personalised exercise programming and support. However, digital technology has been a promising method of providing personalised supportive care over a distance [30]. Digital health technology (also known as eHealth) has existed in health research for several years, though there has been an exponential growth throughout the coronavirus 19 (COVID-19) pandemic. A recent review exploring the feasibility of exercise interventions delivered via telehealth for those living with and beyond cancer highlighted that 6.8% of studies explored lung cancer, whereas breast cancer represented 62% [31]. Thus, it is critical that suitable digital technology is created to support those LWBLC, given the majority of research focuses on those living with and beyond breast and prostate cancer.

Those LWBLC tend to be older individuals (≥ 65y); within the UK, 44% of new diagnoses of lung cancer are those ≥75 years [32]. Given the typical elderly nature of those LWBLC, web-based platforms may increase usability with larger fonts, images, videos, and designs that require less precise mouse manoeuvrability as compared to printed materials or smartphone apps [33]. Additionally, web-based platforms have the capability to deliver personal advice (a.k.a. computer-tailored programmes), educational resources, behaviour change advice, and self-and symptom-monitoring. Notably, these programmes and resources can provide a high-quality and personalised content, promote remote access, minimise travel, and allow the user to maintain a sense of anonymity while maintaining low overall cost [34–36]. Given this ability, it is possible to build on the foundations of movements such as exercise is a medicine and lifestyle medicine [37, 38].

Those LWBLC are more likely to become seriously ill if contracting the COVID-19 virus due to their older age or undergoing chemotherapy and/or radiotherapy, which suppresses their immune system [31]. Given the high symptom burden and the complexity of the treatment-related side effects, such as immunosuppression with chemotherapy agents, there must be an alternative to the typical face-to-face supervised approach. Digital health exercise interventions have previously demonstrated feasibility/acceptability for those living with and beyond breast, gynaecological, multiple myeloma, myelodysplastic syndrome, lymphoma, Hodgkin lymphoma, Leukaemia, non-Hodgkin lymphoma, endometrial, prostate, and metastatic prostate cancer [31, 34, 39]. However, our previously published review shows that online supportive care is in its infancy for those LWBLC, particularly for physical activity focused online supportive care [40]. Our study aims to conduct a feasibility pilot study of a computer-tailored web-based platform, ExerciseGuide UK, which will add to the limited available evidence for those LWBLC. This protocol details the steps taken to adapt an existing platform that has been used for those with a history of breast cancer [41, 42] and metastatic prostate cancer [39] for those LWBLC and describes the methods of the single-group feasibility study. Publication of this protocol intends to increase the transparency of the steps taken to adapt and develop this intervention and develop a more comprehensive understanding of scientific rigour and results.

## Methods

### Study design

This study is a single-group feasibility study. The participants will complete an 8-week web-based computer-tailored physical activity intervention with personalised educational resources. Mixed-methods analyses will be employed with primary outcomes exploring the feasibility and acceptability of the web-based platform. Secondary outcomes examining quality of life, anxiety, and depression will be collected via questionnaires. Physical activity and exercise will be collected weekly via tracking modules, wherein participants can self-report physical activities and exercises completed and any concerns. Fifteen participants will be invited to participate in post-study interviews will be conducted. Interviews will continue if not saturated.

The study has been registered on the ClinicalTrails.gov website [43] (ID: NCT05121259)*,* and ethical clearance was obtained by the Health Research Authority (approval: 21/SC/0174). The reporting of the study protocol adheres to the Standard Protocol Items: Recommendations for Interventional Trials (SPIRIT) guidelines [44]. A participant timeline is presented following the SPIRT guidance in Table 1.

**Table 1:**
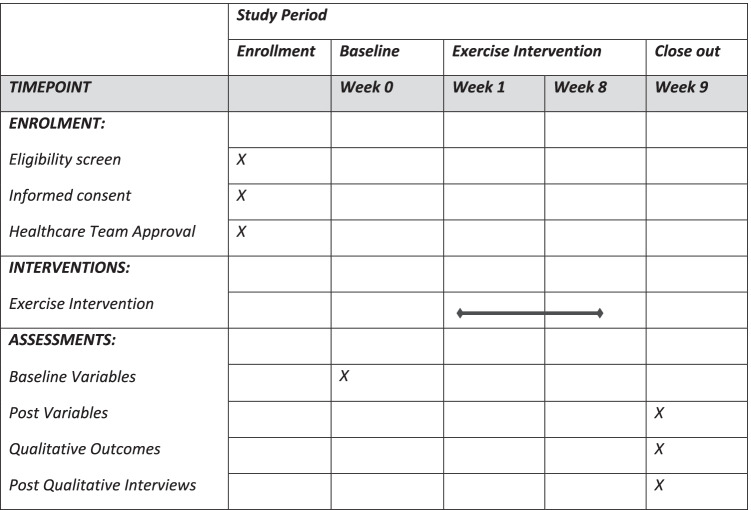
Showing SPIRT figure for the enrolment, baseline, intervention, and assessments

### Study setting

The study is being conducted in Kingston upon Hull, UK. The participants will be identified and recruited via Hull University Teaching Hospital NHS Trust.

Recruitment began in January 2022 and will cease in May 2022 or when the sample size has been reached. The sample size was pre-specified at 35 individuals LWBLC. The sample size is based on a recent systematic review that examined the feasibility, acceptability, and potential efficacy of online supportive care for those LWBLC [40], literature regarding sample size for pilot and feasibility studies [45, 46], and clinical expertise from a senior lung oncology consultant.

### Participants and screening

**Fig. 1:**
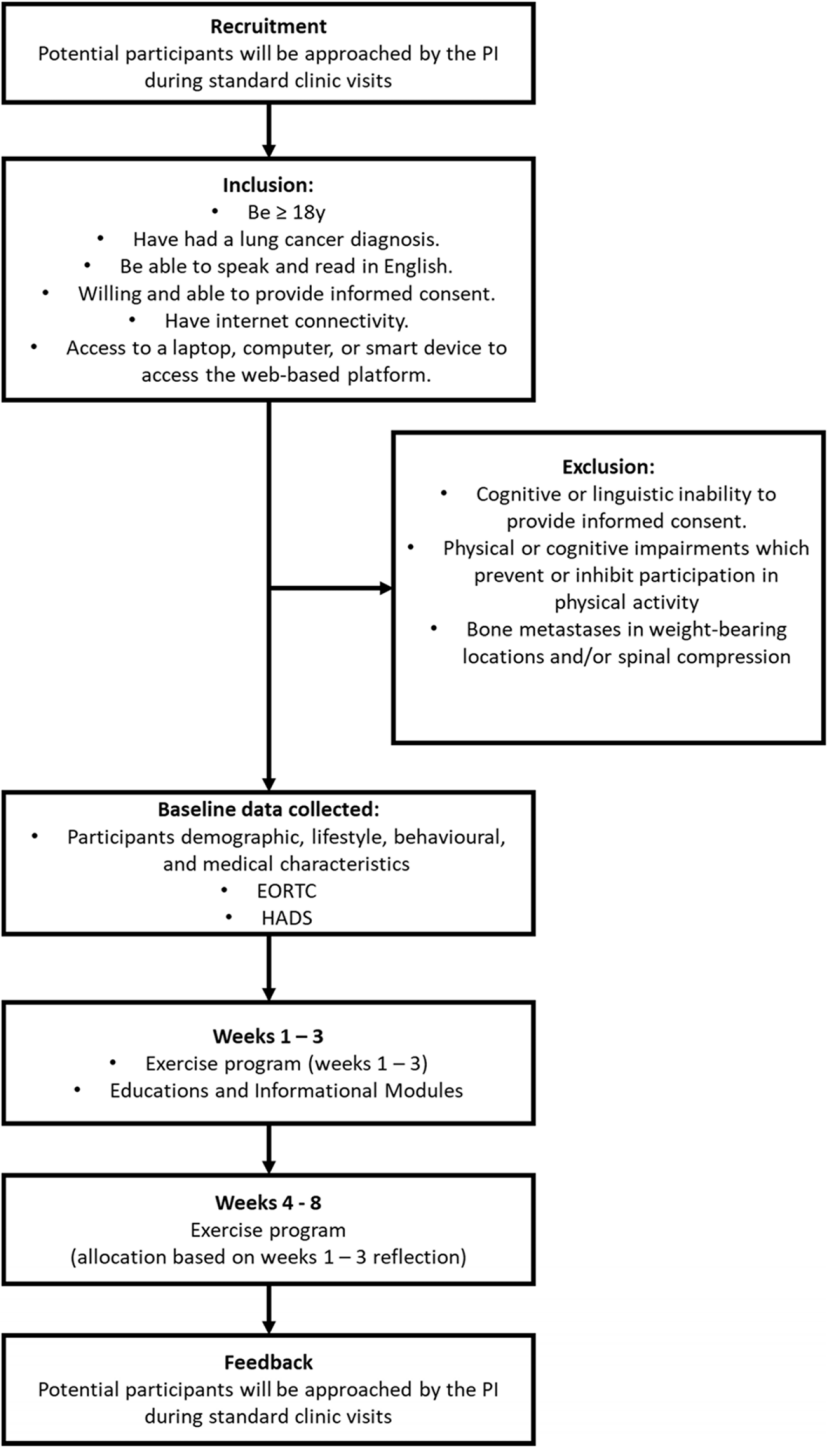
Demonstrating the flow of participants throughout the study

The participants will be recruited through the lung cancer clinic at Hull University Teaching Hospital. The primary investigator will disseminate the recruitment information with assistance from participating site oncologists to those who meet the inclusion criteria during routine appointments. Interested participants will contact a member of the research team to obtain informed consent, provide answers to any outstanding questions, and process baseline data collection. Figure 1 illustrates a flow diagram of participant engagement.

### Eligibility

The participants will be screened against predetermined eligibility criteria and approved for physical activity by their clinician. The participants must have received a lung cancer diagnosis or cancer of the lung (e.g. pleural mesothelioma), either non-small cell lung cancer or small cell lung cancer. In addition, the participants must be 18 years or older, able to speak and communicate in English, be willing to provide informed consent, have access to a smart device that can display the website (e.g. laptop or tablet), and have internet access.

The participants will be considered ineligible if they are under 18 years of age at the time of screening, unable to provide informed consent due to cognitive or linguistic inability, or have a physiological and/or cognitive impairment that would prevent or inhibit participation of moderate aerobic and resistance-related physical activity. In addition, the participants will be excluded if they have identified bone metastases in weight-bearing locations and/or spinal compression, which may inhibit or prevent their safe participation in unsupervised exercise.

### Intervention

#### Intervention development and adaptions

##### Early development

**Fig. 2:**
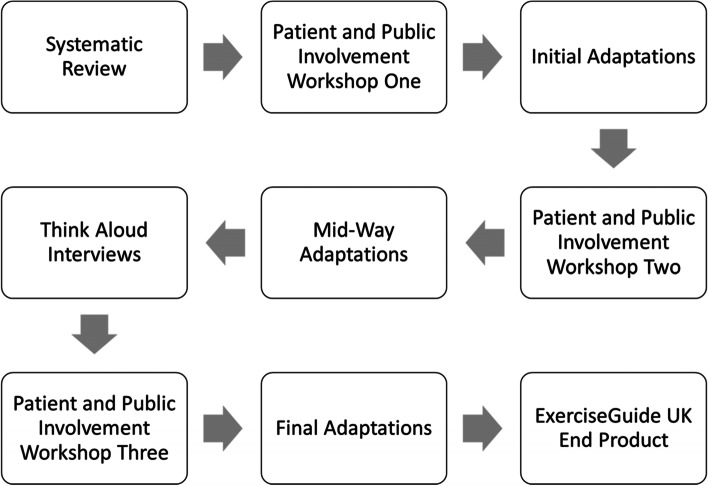
Overview of the process taken to adapt and create new content for ExerciseGuide UK

The original conception of the web-based platform on which ExerciseGuide was built and was developed by Vandelanotte and colleagues [47]. Previously ExerciseGuide has been adapted and used in oncological populations such as breast cancer [41, 42] and metastatic prostate cancer [39]. To ensure the adaption of ExerciseGuide for those LWBLC, an iterative adaptive process was undertaken (see Fig. 2).

A systematic review was initially undertaken to appraise the current literature regarding the feasibility, acceptability, and potential efficacy of online supportive care platforms for those LWBLC [40]. Subsequently, iterative patient and public involvement (PPI) workshops were conducted with volunteers who had experience with LWBLC as a patient, carer of someone, or family member. Think Aloud interviews were conducted with seven participants LWBLC via Zoom. Positive and negative quotes pertaining to each given task were presented in tabular format (see supplemental material 1). A detailed summary of the Think Aloud interviews can be found in supplemental material 2.

##### Final adaptions

**Fig. 3:**
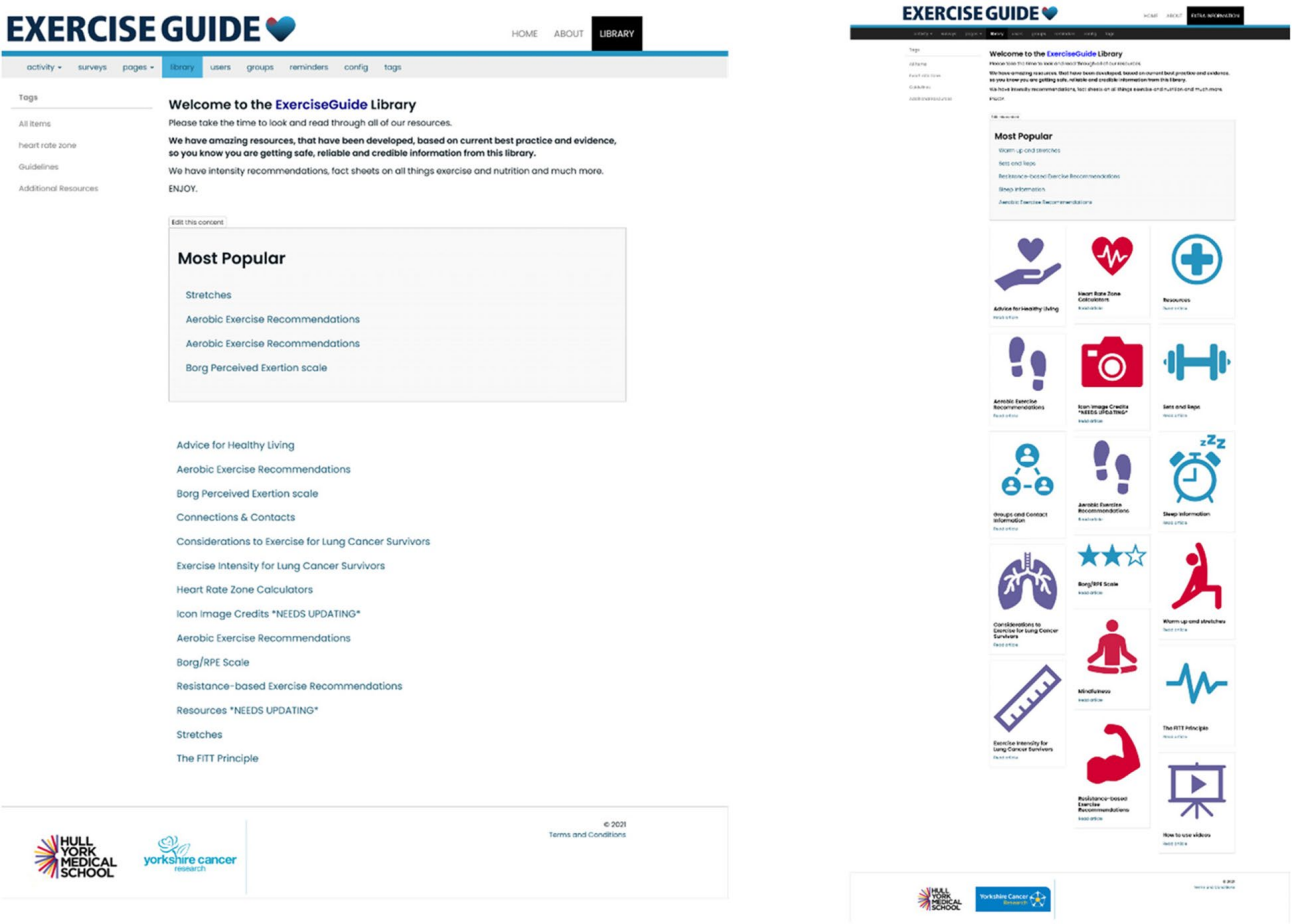
Original (left) and revised (right) screenshots of the Extra Information page (formerly known as Library) on ExerciseGuide UK. Revisions were made based on Think Aloud interviews and agreement with the PPI group

The agreed change was by a mutual consensus with the PPI members. An example of agreed change can be demonstrated within the formerly known library. The participants in the Think Aloud interviews struggled to find a location where extra information would be located and once found, believed the library looked “boring.” Therefore, agreements were reached with the PPI group and research team to change the name “Library” to “Extra Information” and apply image thumbnails to the hyperlinks to provide a more inviting and interesting page (see Fig. 3).

#### Intervention description

**Fig. 4:**
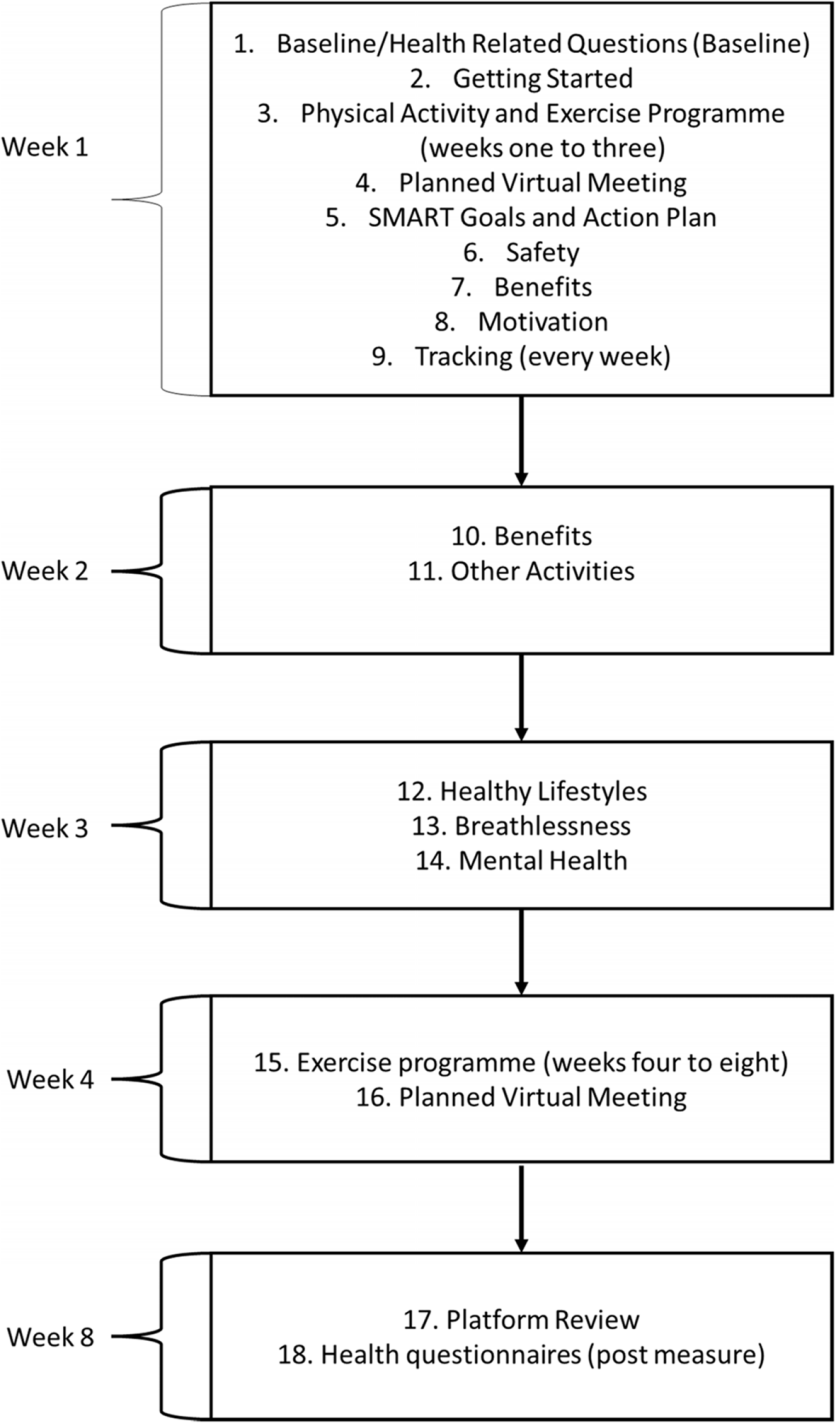
Website flow over 8 weeks

ExerciseGuide UK consists of 18 modules released over 8 weeks. A complete list of the modules can be found in the supplementary material. The website architecture adopts a tunnelled approach, as opposed to free choice. A tunnelled approach allows users to access small batches of information over a pre-specified time and with a predetermined flow [48] opposed to immediate full access [49]. The tunnelled design and module release timings are illustrated in Fig. 4.

**Fig. 5:**
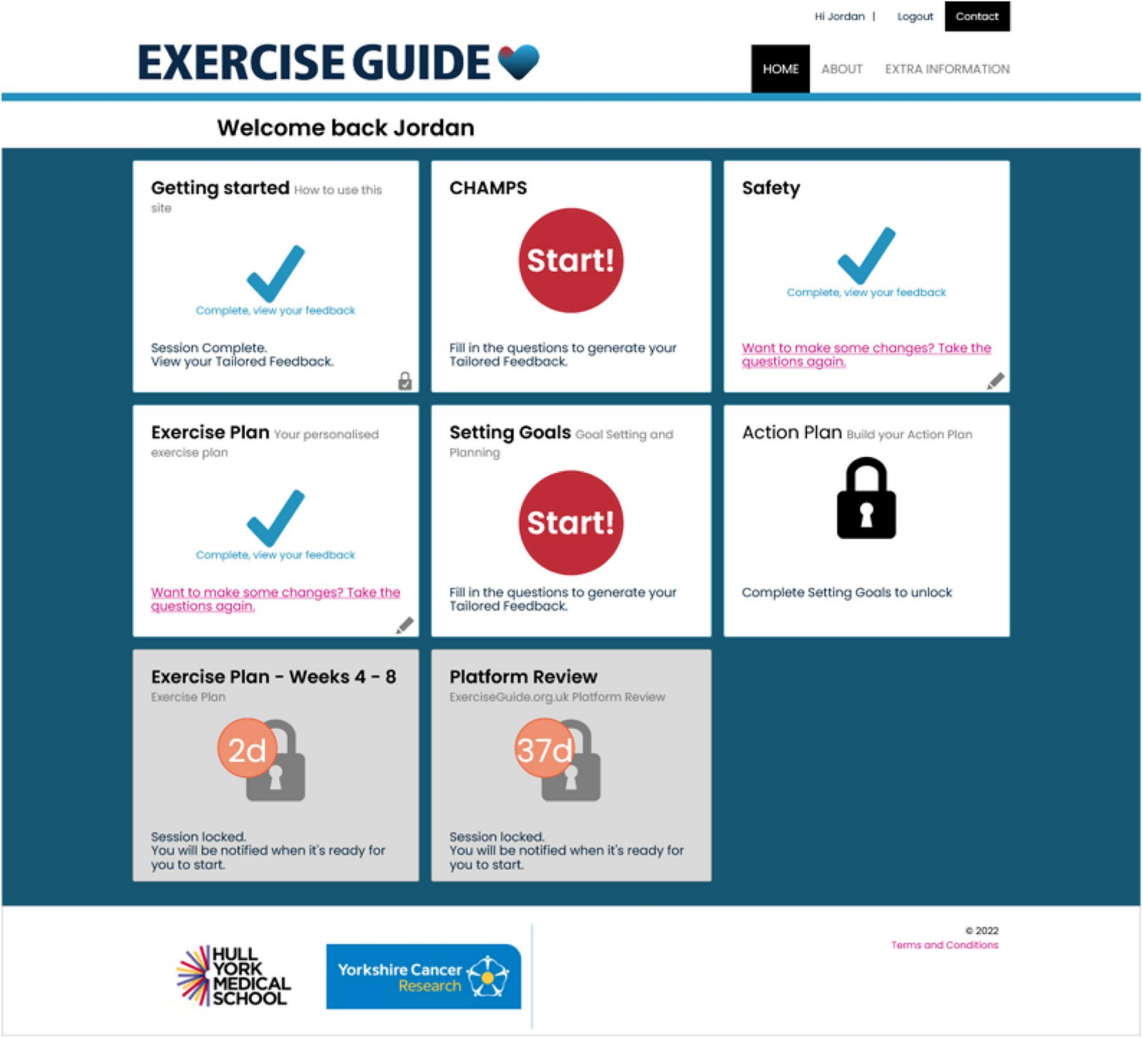
Illustrating an example of the dashboard

The dashboard (shown in Fig. 5) where modules become active throughout the intervention duration. Upcoming modules will be displayed on the dashboard with a countdown until they become accessible.

#### Website tailoring

The website will deliver computer-tailored evidence-based modules, including an 8-week exercise programme and supporting information. The content of the modules will be tailored based on individual participant characteristics through an automated computer process using IF-THEN statements. Modules will contain questions that will have a corresponding message (or feedback item) in the database. Thus, IF a participant answers one or more questions, THEN the corresponding message(s) will appear in the module. Computer tailoring has been shown to increase efficacy and safety of behaviour change and physical activity by delivering personally relevant content [50–52]. An outline of the modules containing feedback items, their tailoring properties, and the behaviour change mechanism of action is detailed in Table 2.

**Table 2:** Illustrating the breakdown of modules, description, tailoring, and mechanisms of action

Module	Module description	Tailoring	Mechanism of action
Getting started	Introductory module to the website. Demonstrating how to use and navigate the website.	No tailoring	● Knowledge ● Self-efficacy
Physical activity programme	Provide a personally tailored physical activity programme in two sections. Section one will cover week 1 to week 3. Section two will cover week 4 to week 8. Additionally, introductory safety information is provided.	Tailoring was based on pre-set questions which covered prior physical activity and exercise experience, physical health limitations.	● Knowledge ● Self-efficacy ● Intentions
SMART goals	Provide information regarding SMART goals. Linked to the Action plan. Participants will set their own SMART goal.	Personalised introduction with messaged based on previous goal setting habits.	● Knowledge ● Goals/behavioural Regulation ● Intentions ● Motivation
Action plan	Supported by the SMART goals module, the action plan guides participants to set a personally relevant and meaning plan to achieve their SMART Goal.	Not tailored. Participants are guided to set an action plan with specific questions. Ultimately setting a personalised action plan.	● Goals/behavioural Regulation ● Intentions ● Motivation
Exercise safety	Provide safety information for those LWBLC regarding being physically active and engaging in exercise.	Further in-depth guidance is provided for specific health- and cancer-related concerns.	● Knowledge ● Self-efficacy ● Beliefs about capabilities ● Needs ● Perceived Susceptibility/vulnerability
Exercise benefits	Provide informative content surrounding benefits of physical activity for those LWBLC.	Health issues and cancer-related side effects which may be improved via physical activity and exercise.	● Knowledge ● Optimism ● Self-efficacy ● Intentions ● Motivation ● Beliefs about consequences
Motivation	Content surrounding motivation, barriers and enablers to physical activity, and habit formation.	Identify and assistive feedback on specific barriers to physical activity and exercise.	● Emotion ● Attitude towards the Behaviour ● Values ● Motivation ● Automaticity
Tracking module	Provides an opportunity for self-monitoring of exercise and healthy lifestyle behaviours and outcomes.		● General attitudes/beliefs ● Self-regulation
Other activities	Covers information regarding what is physical activity, exercise, and physical fitness. Further information regarding non-conventional activities and exercises.	Tailored information provided around methods of getting in “other” types of activities within their daily lives.	● Knowledge ● Optimism ● Self-efficacy ● Intentions
Health lifestyles	Provide informative content, both generally and lung cancer specific regarding lifestyle factors which may increase health-related quality of life.	Tailored information based on treatment type and lifestyle habits (smoking, alcohol, sleep, activity minutes), and personal values	● Knowledge ● Optimism ● Self-efficacy ● Intentions ● Motivation ● Beliefs about consequences
Breathlessness	Provide foundational information of what is breathlessness, causes, and exercises to help (both video and written demonstrations).	No tailoring	● Knowledge ● Self-efficacy
Mental health	Provides an introduction to mental health and lung cancer. Additionally, this module provides multiple links to external sources which discuss lung cancer and mental health-related factors.	No tailoring	● Knowledge ● Signage and support

#### Exercise prescription

The exercise prescription covers 8 weeks of tailored exercise. The adaptions to strength training are generally evident in individuals after 8 weeks [53, 54]. However, some studies highlight an increase in muscle cross-sectional area and strength after 2 to 4 weeks [55–57], likely due to neurological or connective tissue adaptations. Furthermore, 8 weeks of aerobic exercise has been illustrated to significantly improve aerobic capacity among those LWBLC [58].

The exercise prescription has been divided into two main categories: resistance-based exercise and aerobic-based exercise. Both components of exercise are based on participant-provided information. Exercise repetitions (reps) and sets are provided for the weekly programmes. Upon cessation of the week 1 to 3 exercise modules, participants will be asked to reflect on weeks 1 to 3 retrospectively. If too challenging, the frequency and intensity will be reduced. If the participants report the programme as appropriate, the programme frequency and intensity will slightly increase throughout weeks 4 to 8. Finally, if the participants report that the exercise programme was too easy, a larger increase in frequency and intensity will occur. Exercise safety information is provided in the exercise programme, prior to exercise prescription, and as a standalone safety module.

Participants will receive a minimum of two tele-coaching sessions at the beginning of weeks 1 and 4 to review the exercise prescription, safety queries, and any questions. Activities will be prescribed based on questions surrounding physical limitations, pain, and exercise experience.

##### Resistance exercise

**Fig. 6:**
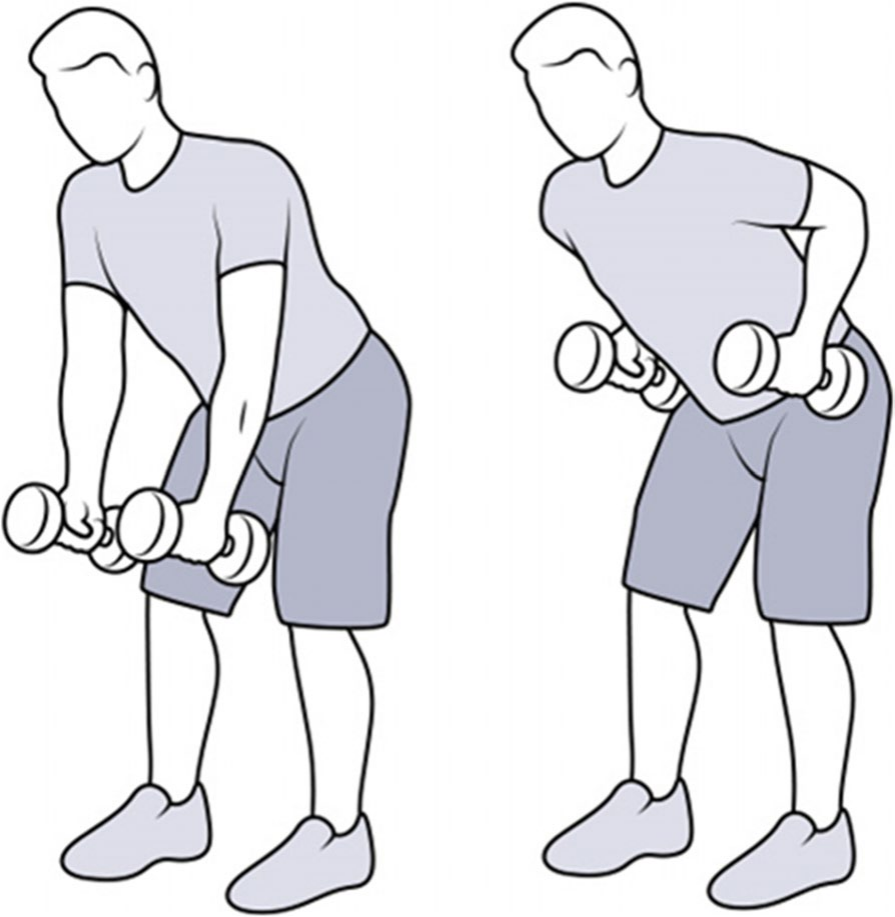
Stills of the animation (via embedded graphic interchange format (GIF) files) of the standing dumbbell row

**Fig. 7:**
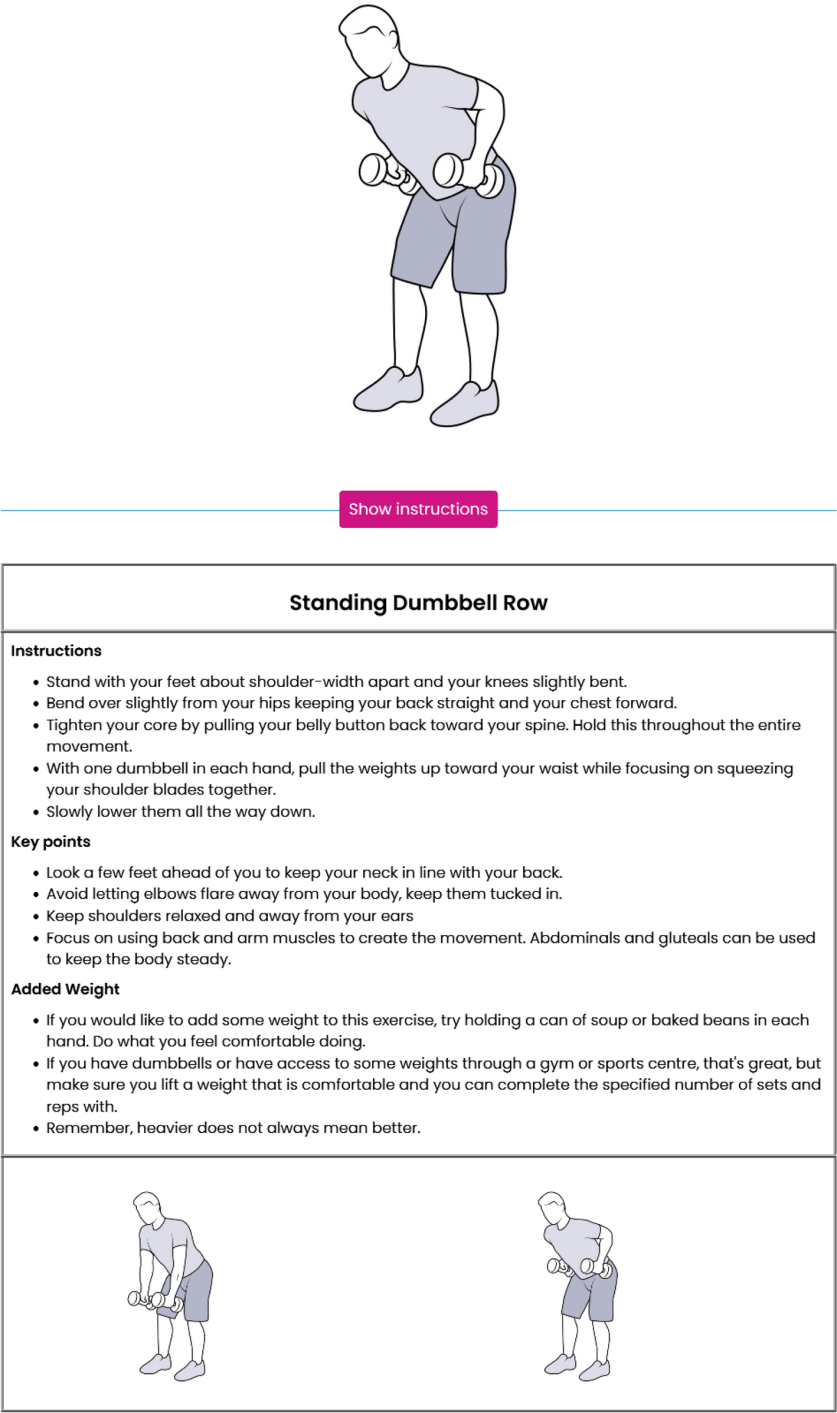
Example of the extra information provided to the participants for the standing bumbbell row

There are twenty-two possible resistance exercises that can be included in the exercise prescription. The computer-tailored website allows tailoring the exercise prescription based on physical activity and exercise experience, current physical activity and exercise levels, and overall and specific limits to physical health and activities of daily living. A list of the exercises is provided in supplementary material 1. An animation demonstration and written instruction will accompany each exercise (see Figs. 6 and 7).

**Fig. 8:**
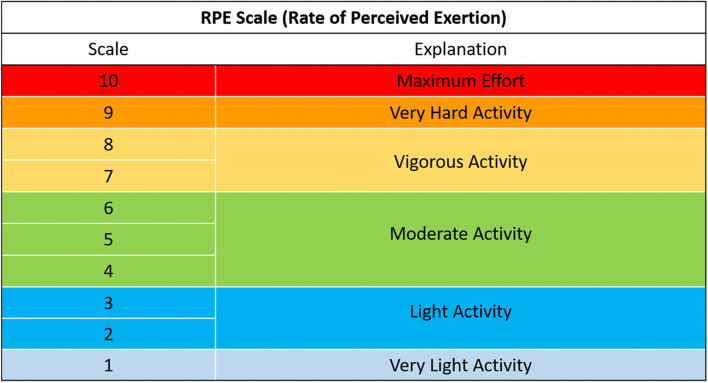
Rate of perceived exertion (RPE) used on ExerciseGuide UK adapted from Borg (1982) [59]

**Fig. 9:**
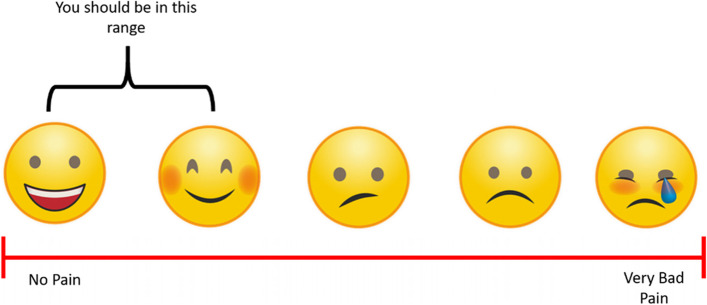
Demonstrating the visual pain rating tool for resistance training exercises on ExerciseGuide UK

The intensity of the resistance exercises will be prescribed with a visual aid tool. In addition, the Rate of Perceived Exertion scale (see Fig. 8) and pain scale (see Fig. 9) will be provided to conceptualise perceived scoring. Participants are informed about aiming for the green zone of moderate activity for the exercises. Information to try contextualising the moderate activity zone will be provided in lay language. For example, referring to body heat and the ability to “talk and not sing,” contextual comparisons which PPI members felt were valuable and understandable.

The frequency of resistance exercises will be 2 days per week for weeks 1 to 3, with eight to 12 reps maximum for two sets. At the end of week 3, the participants will be asked about their experience of the first 3 weeks. If the programme is perceived as “too hard”, the exercise will maintain at a stable level with possible modification throughout. The dose will remain two sessions per week, with two sets and eight to 12 reps for week 4. The participants will be informed to maintain this dose throughout, though at weeks 5 to 8, and they can increase to three sets of any exercise if they feel able.

In contrast, participants who rate the programme easy will be provided with three sessions per week for the remaining 4 weeks with an increased dose of three sets and 8–12 reps. Those who found the programme mildly challenging but achievable will be prescribed 2 days per week for weeks 4 and 5 and 3 days per week for weeks 6, 7, and 8, with reps ranging from 8 to 12. The Borg scale (see Fig. 8) will be used to illustrate the level of perceived exertion for resistance training. The PPI group recommend this scale, limiting the information and scales provided. Moreover, Natio and colleagues (2019) reported the one to ten Borg Scale was useful for elderly non-small cell lung cancer and pancreatic cancer patients performing resistance training [60]. The participants were informed to work at five–eight of their rate of perceived exertion.

The participants engaging in exercise sessions prior to participation in the study will be encouraged to continue their exercise but complete the prescribed exercises from ExerciseGuide UK. The participants will be encouraged by the exercise professional to engage in their own exercise regime in addition to ExerciseGuide UK if feasible.

##### Aerobic exercise

**Fig. 10:**
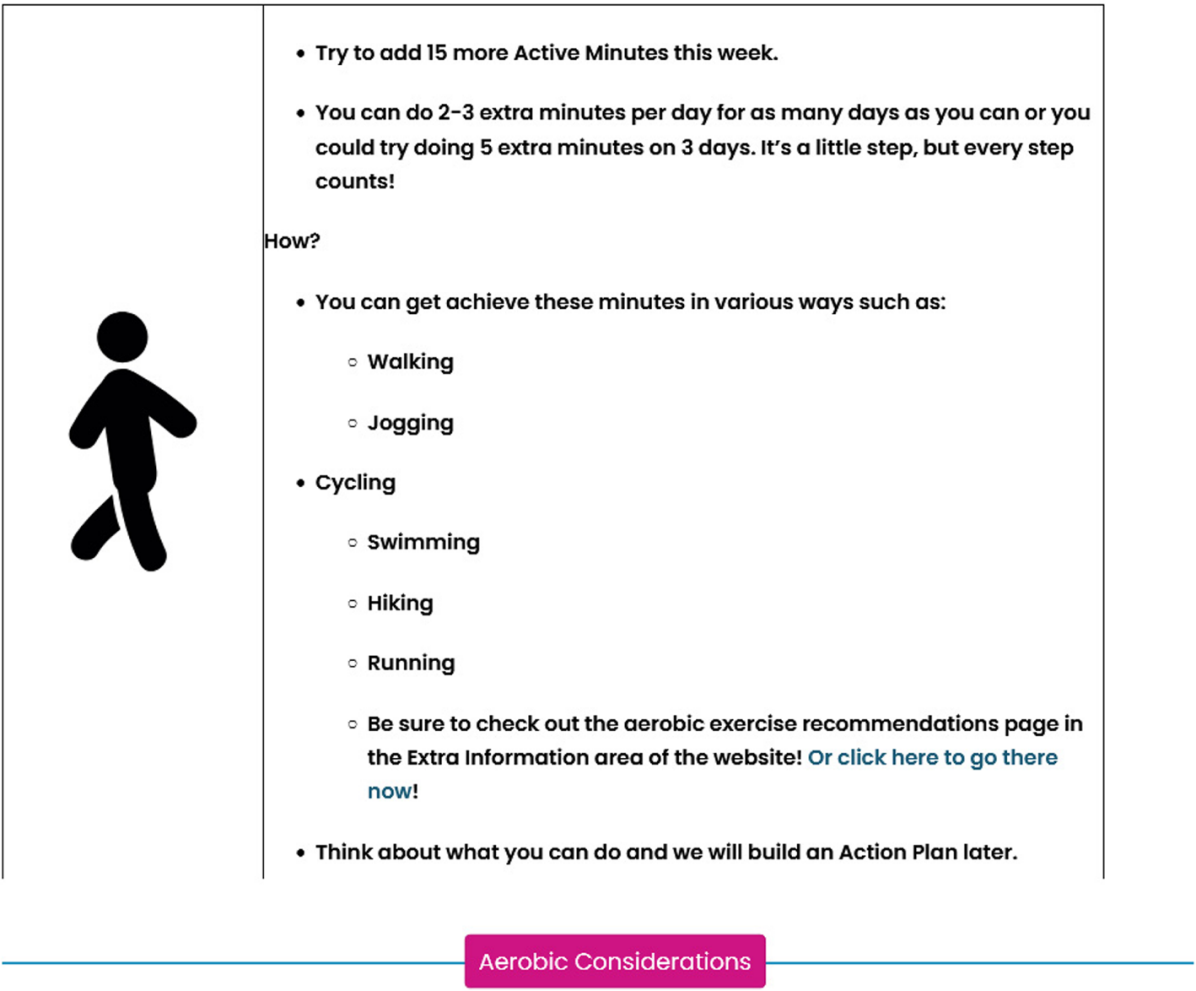
Example-tailored advice for a participant who self-reported 50 physical activity minutes per week. The pink button will release a collapsible drop-down box detailing considerations for aerobic activity, including the FITT principle

Aerobic exercise will be based on their current activity levels. The aerobic physical activity information provided will help participants with suggestions for increasing activity and meeting the physical activity guidelines (if appropriate) for those living with and beyond cancer [13, 61–63]. In addition, the information provided (see Fig. 10) will give examples of exercises with a collapsible drop-down option detailing supplementary information regarding aerobic recommendations and considerations covering the FITT (frequency, intensity, time, and type) principle in detail. Aerobic exercise information is provided based on the participants’ current self-reported aerobic exercise. Using the government guidance of 150 moderate–75 vigorous minutes per week, the participants recorded their current level of aerobic activity. ExerciseGuide UK then illustrated how the participant can increase their aerobic activities in smaller bouts of aerobic activity to reach the recommended guidance [13].

##### Virtual meeting

The participants will meet with one member of the research team at the start of week 1 and week 4 online using video conferencing software or via standard telephone calls. In both meetings, the researcher will walk through the exercise prescription, ascertain the safety of exercises for the participant, and encourage engagement with the programme. The efficacy of behaviour change interventions has been illustrated with human interaction and support, with improvements in adherence and effectiveness of digital interventions [64].

#### Additional intervention components

##### Action planning

ExerciseGuide UK contains a module dedicated to developing an action plan. The action plan within ExerciseGuide UK includes a tunnelled transition following background and introductory information regarding SMART goals. To build the action plan, the website provides examples that help participants think of specific details in relation to being active (e.g. what activity, when, where, how long, and with whom)**.** Upon completion of the questions, the website will offer a structured action plan. The action plan can be modified over the 8 weeks at the user’s discretion and printed off.

Action plans have demonstrated an essential strategy for intended behaviour change by bridging the so-called intention-behaviour gap [65]. Action plans have been included on previous iterations of ExerciseGuide [66] and variations of the computer-tailored platform such as workplace sitting [67, 68]. Furthermore, a systematic review noted that action planning within eHealth interventions has been effective for behaviour change [69].

##### Tracking

An online tracking module has been created to support participants to track their physical activity and general thoughts. The tracking module will provide a mix of open and closed questions regarding aerobic and resistance exercise, health-related fitness, motivation and habits, and general comments. Additionally, the tracking module will provide tailored feedback. This module will be released weekly, though a selection of data (motivation and health status) will be pulled through to subsequent weeks for graphical features (e.g. enablers and health/symptom check-in). Visual aids such as responsive graphs can present past and present data to show progress. The participants will be sent an email reminder upon the release of the tracking module.

##### Extra information

The extra information page acts as a library of cancer-specific information written in layman’s language.

##### Contact form

The website contains an integrated contact form where the participants can email the research team directly. This integrated contact form auto-populations the participant’s name and email for convenience. A copy of the email is sent to the participant’s email they have registered with the platform.

### Measures

#### Feasibility

The feasibility of the study will be assessed via the rate of recruitment and retention over the study duration.

#### Acceptability

The acceptability of ExerciseGuide UK will be assessed using a mixed-methods approach. The participants will be guided into a module with an integrated satisfaction survey upon completing week 8. This survey was modelled of the Systems Usability Scale with questions being tailored to ExerciseGuide. The satisfaction survey was adopted from the Canadian version of ExerciseGuide for those with a diagnosis of breast cancer. In addition, the participants will be provided with the opportunity for real-time module feedback using a five-point Likert scale (1—poor to 5—excellent) and open-ended feedback [70]. Finally, after completing the 8-week programme, 15 participants will be invited to participate in an interview to explore further the satisfaction of the online platform and virtual communication. These will be offered to participants following completion of the study.

#### Usability

In addition to the satisfaction survey will be the Systems Usability Scale [71]. The Systems Usability Score is a 10-item questionnaire with five responses ranging from “strongly disagree” to “strongly agree” [71]. The final question allows respondents to provide further comments in an open-ended format. The criteria for the System Usability Score is ≥ 68% [71, 72].

#### Website usage

Engagement will be assessed using ExerciseGuide UK stored database information and Google analytics integrated website tracking software [73]. The number of times a participant has entered a module answered questions, and tracking log will be counted to establish website and module engagement [74]. Furthermore, Google analytics will be used to examine the time spent on specific modules reading feedback or library articles, as well as to assess non-usage attrition (i.e. the process of participants not using ExerciseGuide UK as intended or at all). Participation in pre-, mid-, and post-telehealth sessions will be noted.

#### Health-related outcomes

Health-related outcomes will be collected at baseline upon registration to the website and immediately following the completion of week 8. The health-related outcomes being assessed pre- and post-study will be the quality of life, anxiety, and depression. Health-related quality of life will be explored via the 30-item European Organisation for Research and Treatment of Cancer Quality of Life Questionnaire-validated questionnaire (EORTC QLQ-C30; version three) [75]. The quality of life domains are divided into multi-item subscales, functional (e.g. physical, role, cognitive, emotional, and social), symptom scales (e.g. fatigue, pain, nausea/vomiting, and dyspnoea), financial hardship, and global health status.

Anxiety and depression will be measured using the Hospital Anxiety Depression Scale [76]. The Hospital Anxiety Depression Scale will measure anxiety and depression using a 14-item scale (seven items for depression and seven items for anxiety). The Hospital Anxiety Depression Scale has been illustrated to show an excellent screening of those with and without clinical symptoms of depression and anxiety to longer questionnaires (MADRS-S and STAI-S) using web-based platforms for those living with and beyond cancer [77].

#### Data management

ExerciseGuide UK website uses a modern framework (CakePHP) that provides a baseline security level. ExerciseGuide UK runs on a trusted hosting platform only over Hypertext Transfer Protocol Secure (HTTPS), using current Transport Layer Security versions. HTTPS is a secure method of sending data between a web server and a web browser. The passwords are never stored as plain text. They will be stored as a salted encrypted hash.

#### Data analysis

##### Pre-established criteria

The feasibility will be assessed based on the pre-established criteria detailed below:

1. The recruitment target of 35 has been reached within the allocated 5 months.
2. Recruitment rate: ≥ 60%.
  - Recruitment rate will be assessed by the number of eligible patients approached relative to the number of participants enrolling in the study.
3. Retention rate: ≥ 85%
  - Retention rate will be assessed as the number of participants who complete 80% of the intervention over the 8-week duration.

Recruitment and retention rates have been established based on a recent systematic review of online supportive care for those LWBLC [40].

Acceptability will be assessed based on the following pre-established criteria:

1. System Usability Score ≥ 68% [71, 72]
2. Positive participant satisfaction illustrated in the end of study survey presented as a mean value on a scale
3. Positive themes identified in follow-up interviews

##### Quantitative data

The quantitative data will be exported to SPSS version 26 (IBM, Chicago, IL, USA) for analysis. Descriptive statistics of the sample and each study measure. Data obtained from the EORTC-QLQ-30 and Hospital Anxiety Depression Scale will be analysed using a paired *t* test (non-parametric, Wilcoxon test). The weekly self-report tracking module will examine adherence to the exercise prescription. Changes in attitudes to physical activity, confidence, and burden will explored post-intervention.

##### Qualitative data

Qualitative data will be obtained via two modalities. Firstly, open-ended questions will be provided in the satisfaction survey at the end of week 8 to elicit qualitative feedback. Any qualitative feedback provided will be exported to a single software for thematic analysis. Secondly, following the completion of the programme, 15 interviews will be conducted and will continue if not saturated. Interviews aim to expand on the quantitative satisfaction questions by exploring prior or current barriers to physical activity and the potential impact (e.g. behaviour and attitude change), usability, and friendliness of ExerciseGuide UK.

All interviews will be transcribed verbatim and analysed using thematic analysis. Using inductive coding, transcriptions will be interpreted, and codes will be generated. The strength of convergence of generated themes will be examined based on the overlapping frequency and range.

##### Sample size

Given that this study is a feasibility study with pre-established criteria for success instead of a primary outcome assessment, a formal sample size calculation is not necessary [46]. The sample size for this study is a maximum of 35 individuals LWBLC. The sample size was based on the previous research into online supportive care for those LWBLC and clinician expertise. Firstly, previous research exploring online supportive care for those LWBLC was considered. A recent review reported a mean sample size of eight studies examining online supportive care for those LWBLC [40]. Of the eight studies, six were pilot and feasibility studies. The mean sample size over the six pilot and feasibility studies was 29 ± 33, which demonstrated satisfactory detection of feasibility and acceptability concerns. Furthermore, literature highlighted 35 participants is satisfactory per group [78]. Secondly, clinical expertise was sought out. Based on the recommendation from a senior academic lung cancer clinician based on the studies specified recruitment period (5 months) based on multiple considerations (e.g. caseload and eligibility criteria). Furthermore, the sample of 35 individuals consulted with statistician regarding to ensure adequate size to determine feasibility issues.

## Discussion

The primary aim of this study is to explore the feasibility and acceptability of ExerciseGuide UK (an online supportive care platform) for those LWBLC. In addition, the publication of the protocol aims to increase the transparency and reliability of the study and methods.

Online supportive care has been a rapidly emerging field in exercise oncology over the past decade, especially since the inception of COVID-19 in March 2020. However, a recent review exploring the feasibility of exercise interventions delivered via telehealth for those living with and beyond cancer [31] highlighted a lack of research for those LWBLC. For example, of the 29 interventions Morrison and colleagues (2020) appraised, 6.9% of interventions were within those specifically LWBLC. Furthermore, no study explored exercise and telehealth for those LWBLC within the UK [31]. Data collected through the International Cancer Benchmarking Partnership has shown that over the past several decades, in countries with similar healthcare systems (Australia, Norway, Canada, Denmark, New Zealand, Ireland, and the UK), the UK has ranked lowest for 1-year lung cancer survival [79]. Thus, highlighting further research is paramount to address the below-average survival rate for those LWBLC.

ExerciseGuide UK provides a unique and novel method of providing those LWBLC with an 8-week tailored physical activity programme and personalised educational resources using distance-based methods. Supervised (in-person) exercise interventions are thought to be superior to unsupervised exercise programmes [29]. However, ExerciseGuide UK provides non-real-time supervision while using a distance-based approach. Though, there is limited existing high-quality evidence for those LWBLC. Those LWBLC often display a higher unmet symptom burden and lower quality of life than other prevalent cancer types [80]. Higher unmet symptom burdens and lower quality of life may lead to physical, psychological, and financial disparities, leading to unattainable or achievable standard in-person exercise programmes. Literature has demonstrated key benefits of digital technology regarding accessibility, reach, and convenience through online or computer-mediated communication because it can mitigate temporal and geographical barriers [81–83]. ExerciseGuide UK can maintain personally tailored content for those restricted by location or schedule.

Though ExerciseGuide UK presents possible benefits for those LWBLC, there are some noted limitations. Firstly, while digital technology can reduce temporal and geographical barriers to interventions, this is dependent on several presumptions. Having access to a laptop, computer, or smart device (e.g. tablets and smartwatches) that would enable participation may be varied. Lung cancer incidence is higher for those living in deprived regions and with lower socioeconomic status [84]. However, reports illustrate that those living in urban areas have higher-speed Internet availability [85] compared to those living in rural. Overall, urban areas tend to be more socially deprived than rural areas [86]. Furthermore, the average cost of fixed data (broadband) and voice packaged monthly costs in the UK was £37.25 [87].

Secondly, research waste is an ongoing concern within health research. Up to 85% of research within health is understood to be wasted due to poor research design, inadequate reviews of literature, and unpublished research [88]. The meaningful involvement of the target population of an intervention may reduce the ongoing concern of research waste [89] while ensuring the intervention is appropriate and meaningful for end-users. ExerciseGuide UK has been adapted using an iterative approach with those LWBLC and their family members and qualitative interviews.

Lastly, recruiting those LWBLC into clinical research can be challenging [90]. The recruitment will occur in a lung cancer clinic with assistance from hospital oncologists.

## Conclusion

ExerciseGuide UK provides a unique and novel approach to providing tailored physical activity programmes and educational resources to those LWBLC. However, there is limited high-level evidence within online supportive care for those LWBLC. Thus, the feasibility study exploring ExerciseGuide UK will provide insight into usability concerns that may be revised prior to larger-scale trials, potentially reducing research waste [91]. Building on the evidence collected as part of the feasibility and acceptability trial, the authors plan to revise the website and explore methods of facilitating digital technology usage within lung cancer, comparative assessment of those using a digital physical activity tool vs standard care, and further development of physical activity and health advice (e.g. nutrition).

## Supplementary Information

**Additional file 1:** Table of change

**Additional file 2:** Think Aloud Interviews

## Data Availability

Not applicable

## Abbreviations

LWBLC: Living with and beyond lung cancer
PHP: Hypertext pre-processor
ACSM: American College of Sports Medicine
COVID-19: Coronavirus 19
RCT: Randomised control trail
PPI: Patient and public involvement
SMART Goals: Specific, Measurable, Attainable, Realistic, and Timely Goals
EORTC: European Organisation for Research and Treatment Of Cancer
SPIRIT: Standard Protocol Items: Recommendations for Interventional Trials
