# Supplementary material for: Evaluating a web-based computer-tailored physical activity intervention for those living with and beyond lung cancer (ExerciseGuide UK): protocol for a single group feasibility and acceptability study
Source: Pilot Feasibility Stud. 2022 Aug 13;8:182. doi: 10.1186/s40814-022-01129-6 (PMC9375062; doi:10.1186/s40814-022-01129-6)
Supplement: Supplementary file 1 — Additional file 1: Table of change [file 40814_2022_1129_MOESM1_ESM.docx]

**Supplementary Material Two**

# Supplementary Two: Table of Change

| **Negative Comments** | **Positive Comments** | **Possible Change** | **Reason for Change** | **Agreed Change** | **MoScoW** |
| --- | --- | --- | --- | --- | --- |
| *Signup Page and Getting started page* | | | | | |
|  | “I think it's [the sign up/sign up page] really quite clear. Yeah, it gives me an option to get started now. Or, if I'm already registered. I can log straight in. I'm totally drawn to the it's free. In that. So yeah.” – P005 | N/A | N/A | N/A | N/A |
| “You going straight into the getting started and you had that presumption that. Everyone has come to this resource. Specifically for the exercise. Not that they’re just a little curious. So why didn't you know, sell it with the benefits at the start” – P001  “Improved well being. I haven't seen much of that being mentioned at the beginning, I think. Maybe do a bit of selling at the start the front end, but yes, that is a nice idea” – P003 |  | Add some information regarding benefits of physical activity to the introductory page | EAS, REP, EXP (via PPI) | Add in some general and lung cancer specific benefits to the introduction module. | MUST |
| “And I can't see finish” – P001  “Do you want me to click on action plan?” – P003  “So. It says please click finish in your bottom right to continue. I can't see that.. it's got action plan there in the bottom right. So is that where I'm going?” – P005 |  | Change the button which says “Action Plan” to “Finish” | REP, EAS | Change the button which says “Action Plan” to “Finish” | MUST |
|  | “Yeah, I do think it's quite a good idea. Just in case people aren't, you know, would rather do visual than reading.” – P005  “Yeah, I like the idea videos coz I like. I think yeah. By seeing. Like a visual learner, I think I'm all. Yeah I like to see what someone is doing rather than just read. What I'm meant to do? If you can see the video, it's easier to know we supposed to be doing. And you last likely to make mistakes.” – P007 | N/A | N/A | N/A | N/A |
| “It looks inviting, yeah. It looks inviting it doesn't. It doesn't look specific to lung cancer, so I might be thinking, Oh my gosh is it people like? Is it doing during a triathlon? Who are signing up for it? Do you know what I mean? And would that be way out of my league?” – P007 |  | Add the phrase ‘living with and beyond lung cancer’ onto the sign-up page | EAS, EXP (via PPI) | Added the phrase ‘who have had a lung cancer diagnosis’ | SHOULD |
| “I'm having to lean forward and struggling to read it [the dashboard image].” – P003  “Yes, it's a little small. So yeah. So the screen I'm looking at. It seems to be yeah, quite small and difficult to read.” – P004 |  | Make image larger | EAS | Increased the size of the images on this module. | MUST |
|  | “yeah, that's nice, so description. Think it is a compass …yeah, so this is a type of thing. That when I was diagnosed I would have quite liked. You know, cause I did ask a lot of questions. And they were not really met very clearly. Or with a lot of sort of confusion” – P004 | N/A | N/A | N/A | N/A |
|  | “I think. Yeah. It's it's easy to read, it's nice. It's a nice size font and text. Yeah.” [getting started module] – P005 | N/A | N/A | N/A | N/A |
|  | “Ohh, I love videos. That's great. Really relieved, now. I've got to here [Participant is referring to the video in the getting started module.] because I'm thinking. Information, information information. Will I remember this? Did I need it? But now I've seen this and that's wow that's great.” – P006 | N/A | N/A | N/A | N/A |
| “Maybe for someone like me it might be helpful. So my up here. You we have got videos to support you. Because I might have stopped scrolling down to be honest and gone into one of these things before I realised there was a video. I think it's quite important” – P006 |  | Let people know there are videos further down? | NC, EXP (via PPI) | The detail regarding videos below was not provided earlier. | N/A |
| *Physical Activities and Exercise* | | | | | |
|  | *“*I think this is a really good without even reading the questions. The bullet you can click on. When someone's looking at that, they can rattle through there very quick. And see what they're doing sort of thing” – P002 | N/A | N/A | N/A | N/A |
|  | “I mean, the visuals are good, they're very clear in terms of what you need to do” – P002  “And the animations good. It's a good size.” – P005  “You know what I like? The animation is very simple. There is sometimes you look at YouTube for example. They talk about the call and they give you far too much information. Well, that's really, really simple. Which is good. I'm not being blinded by science here” | N/A | N/A | N/A | N/A |
| “Again, I think that could be, I think that could be a bit bigger” [aerobic walking advice] – P005 |  | Increase the size of the aerobic advice | EAS | Increased the size of the table | MUST |
| *“*We ask a couple of questions. Are there any limitations which you may have? So we ask a couple of quick questions. Please answer the following eight questions. Well, are there two questions or eight?” – P007 |  | Change “couple of quick questions” to “some quick questions” | EAS | Changed “couple of quick questions” to “some quick questions” | SHOULD |
| *“*I am not sure, or understanding what it is you’re asking, I am guessing, you are asking what I do at the moment or are you asking what experience I have had in the past” – P001  *“*How would you rate? Erm. I don't know with how would you rate your exercise experience. Erm. What does that I mean? I'm not, yeah, I'm not understanding that really. As in my experience with equipment in the gym. I don't know” – P005 |  | Revise question to include the terms “past and present”.  Additionally, add an example. | EAS, EXP (via PPI) | Revised to state “How would you rate your current physical activity and exercise ability?” and added an example. | MUST |
| *“*I guess that is now and erm, probably needs some more clarification, as in do we class walking as exercise, or do we class erm, more than walking, like in a gym or doing yoga or something” – P001 |  | Add in some examples | EAS, EXP (via PPI) | Added in a range of examples, including lighter activities. | SHOULD |
| “Another thing that's missing here is what's the benefit? If I was someone who picked this up and was told to do a standing dumbbell row, what am I getting out of it” – P002 |  | Add in per exercise what is the benefit | NC, EXP (via PPI) | No change. PPI group voiced concerns this may over complicate the currently clear information. | N/A |
| “And then the same again. I guess. There's no instructions on this one.” – P002  *“*Oh, this button doesn't seem to be. Is it coming up?” [referring to the Triceps kickback] – P003  *“*I'm doing the. Show instructions but they're not coming up.” – P005 |  | Remove bug preventing show instructions not working on multiple exercises. | EAS | Removed bug, show instructions now work for all exercises. | MUST |
|  | “Yeah, I like that building up as you go through” – P003 | N/A | N/A | N/A | N/A |
| *“*So along the scale it hasn't got a number, so it's difficult to use” – P004 |  | Provide numbers along the bottom | EXP (via PPI) | Provided numbers along the scale | COULD |
| *“*maybe it should be a little larger” – P004  *“*The actual button you can click on could be a bit bigger or maybe.” – P005 |  | Increase the size of the show instructions button | EXP (via PPI) | Increased the size of all show instructions for resistance exercises and changed colour to pink to increase awareness | SHOULD |
|  | “There's a good range of exercises. Yes, fairly clear and straight forward” – P004  “I think the instructions are really clear. Erm, I like, I like how it's out there with the instructions and then the key points is really good.” – P005  “Yep, that's good and clear” – P006 | N/A | N/A | N/A | N/A |
| *SMART Goals and Action Planning* | | | | | |
|  | *“*Right so yeah. I mean, yeah, it's easy to understand. And yeah. I can press finish.” – P001  “Yeah. Yep. Yeah, quite like that, it's quite nice” – P003  “Well, I say. Okay. Okay, that's quite clear, yeah. Okay, yeah… Yes, it's clear, Yep.” – P004  “yeah, but I like the. The responses my responses are in bold. That makes it quite clear… Or print this out, so that's good.  ” – P005 | N/A | N/A | N/A | N/A |
| *“*The only thing I would say you know. Having a calorie restriction as a risk. I don't know if it's not day and age, is that considered to be healthy or not?” – P002 |  | Replace calorie specific goal example with another healthy lifestyle focused goal | EXP (via PPI) | Reinforced the physical activity theme with another physical activity SMART goal example. |  |
| *“*That's a strange way of saying, isn't it about your goal setting habits… Would it be simpler, are now going to ask you too. Going to ask you a few questions. To help you set your goals.” – P003 |  | Revise question to “we are now going to ask you about your goal setting experience” | EXP (via PPI) | Revised to " We are now going to ask you a few questions about setting goals in relation to exercise.” | SHOULD |
| *“*Yes, that [Q1] shouldn't be yes or no, should it?” – P003 |  | Change to Yes, sometimes, no? | NC, EXP (via PPI) | No change based on discussion with PPI members. | N/A |
| “So for your overall goal, I would think your overall goal was I would like to be eating 2200 calories per day and two pieces of fruit and veggies snacks regularly by the 15th by the 15th of June. So I would say that the overall goal” – P007 |  | Identify which goal is the generic non-SMART goal and the SMART goal below. | EAS, EXP (via PPI) | Labelled the generic non-SMART goal and the SMART goal for clarity and readability. | MUST |
| “I'm not really sure about this box to be honest” [the types of exercises question] – P003 |  | Add extra information tab detailing examples of activities | EAS, EXP (via PPI) | Added extra information tab detailing examples of activities discussed with the PPI group | MUST |
| “Is it the show extra help? Boxes, are they dynamic or are they static?  …Yeah, I mean I said. If I've said I want to increase my body strength. Walking is not necessarily going to do. It's probably not the best. I need to do sort of weight and resistance and stuff” – P003 |  | The extra information boxes change based on the information provided by the participant throughout the website. | NC | Not currently feasible. | N/A |
| “So you have a box that's not clear. For this module you are aiming. Okay. Maybe another prompt to say where you put your goal in” [the box to write your goal in] – P004 |  | Create a placeholder to guide participants and created a darker and thicker boarder. | EAS, EXP (via PPI) | Created a placeholder to guide participants and created a darker and thicker boarder. | SHOULD |
| “it says we mentioned a specific method, where was that mentioned or have I missed it?” – P005 |  | Remove the statement detailing prior mentioned goal setting content. | EAS | Removed the statement detailing prior mentioned goal setting content. | MUST |
|  | “Yes, it's quite nice [SMART goals infographic], different colours. Sort of stand out a little” – P004  “Okay. Er. Like the colours on that, it's nice [SMART goal infographic].” – P005 | N/A | N/A | N/A | N/A |
|  | “that's good -extra help button on action plan], yeah. Because I wouldn't over remembered” – P005 | N/A | N/A | N/A | N/A |
| “I'm trying to press the enter button but it's it falls under but it's not letting me I can only go on” – P001  “Oh, then I want to. I want to return. To put the M underneath, but it's not letting me… So I want to set out the same as above, but I can't because of the textbox.” – P005 |  | Edit code to allow return function in text area. | REP | Revised website code to allow return function in the text area. | SHOULD |
|  | “And it's good that I've got your examples. It's definitely good that I've got your examples” – P005  “I think it's definitely good to have the examples. To help you along... It's really quiet. It's really quite good advice” – P005 | N/A | N/A | N/A | N/A |
| “Right at this point, I would leave the site. Because I I've got nothing inspiring me I. I would love to. Eat healthier I would love to. Most importantly, exercise. Tell my condition. But this is all intellectual. It's asking me to know to. Although I guess I could type that in. But I haven't seen anything yet that says to me this is different. This will grab you. This will enable you. Not the first time, but you know since diagnosis. Give me some inspiration. You know, maybe I can get to with this programme” – P006 |  | Insert content to illustrate how this platform is different and the benefits which can be gained. | NC, EXP (via PPI) | Inspirational content added to the getting started module was deemed sufficient. | N/A |
| *Accessing Library and Connections and Contacts Page* | | | | | |
| *“*Library. Hmm, erm. That was. I would not normally go to library. It was only my brain trying to think it through so…The probably should be something on the front saying useful information or something.” – P001 |  | Change to supporting information | EAS, EXP (via PPI) | Revised ‘Library’ to ‘Extra Information’ | MUST |
| “I think it could be more pleasing to the eye” – P001  “I mean, the only thing is it's very quiet, isn't it?” – P002 |  | Redesign layout with mirrored layout to dashboard | IMP, REP | Mirrored dashboard to confusing. Inserted image header to all hyperlinked pages, | MUST |
| “Support group OK, there's nothing. Or this is the homepage, isn't it? I probably go to about or maybe the library. I don't know really where to go. Help maybe help. Oh no. So it wouldn't help. It's not help, I'd go into about. It's about the exercise about. It's about the people involved. There's nothing there. Library” – P007  “Erm. There's nothing jumping out with me right now. Like bonus content type of thing.*” – P002*  “I don't think I would have found it without your help. You know what I mean? I can see how you would go into the library” – P007 |  | Revise the name of ‘Connections and Contacts’ to ‘Support Information’. | REP, EXP (via PPI), EAS, IMP | Revised the name of this page to ‘Groups and Contact Information’ with a thumbnail image. | MUST |
| *Help and Contact Us* | | | | | |
| “But that one probably won't be. I'll go to the about page.” – P001  “Usually on their website they will be a contact us at the bottom. but maybe in this one it will be in the about.” – P002  *“*For a technical problem, possibly it depends what the problem is. But if I just wanted to contact you, ask you a question. I wouldn't think of going to help” – P003  “As you can see, it wasn't the first place I went to. Because I went straight to home. Thinking that might be. Something on there? And then I went to about…. I don't know a contact. At the top” – P005 |  | Insert contact information onto the About page. | REP, EAS, EXP (via PPI) | Inserted hyperlinked email contact information and hyperlinked guidance to the help page.  Furthermore, changed ‘Help’ to ‘Contact’ | MUST |
|  | “I mean, it seems fairly straight forward. Your text box. You could say whatever you want” – P002  “Yeah, yeah, that's really good” [the message function] – P005 | N/A | N/A | N/A | N/A |
| “I mean, it's nice providing this form, but it has that drawback that it's gone.” – P003 |  | Send carbon copy email to the participants registered email. | EAS | The platform will send a carbon copy email to the email address the participant has registered on the platform. | SHOULD |
|  | “Yeah, I think that's really good [the pre-populated email field]. It's good because what you often see isn't it.” – P006 | N/A | N/A | N/A | N/A |
| *“*Yeah. I mean the other thing I'm thinking about is chat. You know when the chat things come up. That might be helpful. If someone is getting stuck” – P006 |  | No possible change at the point. | NC | No change. Possibly in future pending size of team. | COULD |
| *Feedback/Tracking* | | | | | |
|  | *“*That's quite good [the tracking module]. You know it gives people to. Keeps people engaged” – P002 | N/A | N/A | N/A | N/A |
| “if you say it may be useful to write down some thoughts, on the aerobic exercises, exercises you did this week, so what ort of thoughts are you wanting me to write?” – P007 |  | Added an extra information tab which gives two examples of thoughts. | EAS, EXP (via PPI) | Provided extra information tab with specific aerobic exercise examples. Examples were discussed with a dedicated PPI group of those caring for or LWBLC. | SHOULD |
| “Muscular strength. How would I rate my muscular strength? I don't know what to put there either” – P007 |  | Added an extra information tab to provide some examples and questions to help guide participants. | EAS, EXP (via PPI) | Provided extra information tab with specific strength exercise examples. Examples were discussed with a dedicated PPI group of those caring for or LWBLC. | SHOULD |
|  | “That's good, having the calendar you can just click on” – P005 | N/A | N/A | N/A | N/A |
|  | “This [the feedback] is, yeah, this is nice. Yeah. You get like a report. Yep, that's good” – P005 | N/A | N/A | N/A | N/A |
| *Explored Other Modules* | | | | | |
|  | “I would take that as in seven days they will unlock” | N/A | N/A | N/A | N/A |
|  | “I like the video demonstration. That would be great.” – P001  “That all looked good. Good information, good diagram, good video so. So yeah.” P001  [Breathlessness module] | N/A | N/A | N/A | N/A |
| “When I pressed on the video, I didn't want to watch it all. And then I didn't know how to come out of it. That was the only thing.” |  | Provide written instructions of video controls. | EAS | Provided written instructions with supplementary figures to illustrate video controls. | SHOULD |
| *“*If I haven't thought of after the first week. I haven't been motivated by the 2nd week. I'll probably give up. And not go back in again. So yes, I think it should be sooner. [motivation module]” -P001 |  | Reduce the time delay of the motivation module from seven day to three days | EAS, EXP (via PPI) | Reduced the time delay of this module from seven days to zero. | MUST |
|  | “yes, then I suppose when not overwhelmed with all different modules. Yeah, because if there was loads of modules, yeah. I think that's a good idea, actually, yes. Yes, because sometimes it can be overwhelming” [staggered release of modules] – P001  “Oh no, definitely, definitely. Something to keep people engaged and get them coming back” – P002  “A staggered approach is good. Yes… You want to keep them interested. You want to keep them going” – P003  “No, yeah yeah, I think it's. It's a good idea, really. Build it up” – P004  “I think staggered. Yeah, because otherwise. It's gonna be information overload” – P007 | N/A | N/A | N/A | N/A |
| *“*One of the questions. How many hours a night do you get sleep? Do you do anything to help people get more sleep? Is there anything sort of showing how to get a better night's sleep?” – P001 |  | Provide more in-depth content on sleep in the Healthy Lifestyles module | EAS, EXP (via PPI) | Maintained summary tailored sleep content with a dedication sleep page in the Extra Information page. | SHOLD |
| *“*I mean, would safely not be best at the start” – P002 |  | Reduce time delay to seven days for the safety module | EXP (via PPI) | Increased the initial safety content in the exercise plan module and delayed the safety module to seven days. | MUST |
| *“*This is quite a strong this question [smoking habits]. And as much as. It goes back to the stigma. That's a smoking disease. Should it be the first question” – P002 |  | Swap questions regarding cancer treatments received (Q2) with smoking habits (Q1). | EAS | Create an introductory message and replaced first question with a question regarding cancer treatments received. | SHOULD |
|  | “It's all good. They're all good questions” – P002  “So yeah, that was. That was very useful, yeah” – P004  [Health lifestyle module] | N/A | N/A | N/A | N/A |
| “But what's this benefits of physical activity? Are you going to be? I'm just wondering why that's down there. Shouldn't you be plugging the benefits? Early on maybe straight away…But you do need to get them in hooked in in the 1st place, don't you? So you need to sell some of the benefits upfront, like. Main benefit for me. Was working on fatigue. Almost all of us cancer patients. Fatigue is the thing. Consistent. Over 100% of her sort of thing” – P003 |  | Reduce the time delay for the Benefits of Physical Activity module to seven days |  | Added in general and lung cancer specific benefits (with hyperlinked literature) to the introductory modules and reduced the time delay to seven days. | SHOULD |
| “I'm just wondering on this sort of thing. I've tried it myself. And not done very well with it. But would there be anything on there with mindfulness and stuff?” – P005 |  | Integrate mindfulness into the prescription | NC | No change at the current stage. Mindfulness is a future direction. | WOULD |
| *“*And there's nothing in here that's making me. That's grabbing me or inspiring me. It's all logical to all rational. But not getting me emotionally. And I think it's a real conundrum” – P006 |  |  |  | Hook, emotional hook? Inspiration? |  |
| *“*Maybe some examples of what they [cues and triggers] could be” – P006 | “OK, good. I'm instantly thinking this is what I need… I really like that we are really excited about you and your interests. I reckon we need to have that blaring earlier” – P006  “I think I think this is really good. Because it is all about. Forming a new habit.” – P006 | Add an example of a cue and a possible response | EAS, EXP (research team) | Added an example of a cue and the associated actions/responses within the module under ‘Habit Formation’ | SHOULD |
| *“*Yes, I'm tilting my head. I'm wearing bifocals and that's really small… It's just a bit small” |  | Increase the size of the enabled and barriers figure | EAS | Increased the size of the enabled and barriers figure | MUST |

Note: IMP: Important for behaviour change; EAS: Easy and uncontroversial; REP: Repeatedly; EXP: Experience; NCON: Does not contradict; NC: Not changed; MoScoW: Must, Should, Could, Would.

# Three: List of possible exercises

- Standing Bicep Curl
- Standing Row
- Standing Shoulder Press
- Standing Chest Press
- All fours with Single Leg Extension
- All Fours Progression
- Seated Knee Extension
- Sit to stand
- Squat
- Standing Calf Raise
- Superman(unilateral)
- Superman (both)
- Side Leg Raise
- Bridge
- Crunches
- Standing Triceps Kickback
- Lunges
- Press up (full)
- Press up (knees)
- Counter Push Up
- Alternating Leg Lowers
- Heel Touches
