# Supplementary material for: Evaluating a web-based computer-tailored physical activity intervention for those living with and beyond lung cancer (ExerciseGuide UK): protocol for a single group feasibility and acceptability study
Source: Pilot Feasibility Stud. 2022 Aug 13;8:182. doi: 10.1186/s40814-022-01129-6 (PMC9375062; doi:10.1186/s40814-022-01129-6)
Supplement: Supplementary file 2 — Additional file 2: Think Aloud Interviews [file 40814_2022_1129_MOESM2_ESM.docx]

**Supplemental Material**

**Think Aloud Interviews**

Think Aloud interviews were conducted with seven individuals LWBLC (five females, two males) via Zoom (Zoom Video Communications; version 5.7.0). The Think Aloud interviews followed the guiding principles delineated by Sharp et al. (2007) (1). In essence, Sharp et al. (2007) state that participants should attempt to speak aloud all their actions and thoughts in real-time. Though, as tasks become increasingly demanding, the rate of silence increases. Sharp and colleagues suggest that if extended silence occurs, the researchers may interrupt participants and remind them to think aloud. Furthermore, interviews should be recorded, fully transcribed verbatim, then recorded by the researcher before analysis.

All participants had stage IV lung cancer. The cohort's mean age was 58y ± 6.2, from four different regions in the UK. Participant characteristics for the Think Aloud study are shown in Table one (see supplementary material two).

Participants were introduced to the ‘Think Aloud’ technique, provided a demonstration by the researcher, and given an introductory activity. Following the introduction, participants were provided access to ExerciseGuide UK through a signup bypass. Participants were asked to complete seven main tasks (agreed with the PPI group). The tasks covered the following:

1. Thoughts on the sign-up page and completion of the getting started module
2. Exercise screening and thoughts on exercise prescription
3. Completion of the SMART* goals and Action Planning modules
4. Accessing the Extra Information (formerly known as Library) and locating the information pertaining to support groups and contacts
5. Accessing and using the Contact (formerly known as Help) function
6. Provide thoughts on the Tracking and Feedback module.
7. Exploring module of interest (time permitting)

*Specific, Measurable, Attainable, Realistic, and Timely.

Positive and negative comments are detailed in the Table of Change (see table XXX), with proposed and agreed changes.

Following the interviews, participants completed the Systems Usability Scale via Qualtrics. ExerciseGuide UK was rated ‘Excellent’ four times, ‘Good’ one time, ‘OK’ one time, and ‘Awful’ one time. The ‘Awful’ rating was the overall result of a participant who reported low digital literacy and limited engagement with digital technology. The majority of participants (5/7) stated they would feel confident using ExerciseGuide UK, and six participants stated they imagine most people would learn to use the platform quickly. The summarised System Usability Score data can be found in table one, with a further breakdown per question in table two. The individual breakdown of usability score are presented in supplementary material two with a breakdown per question in table three.

Table 1: Participant characteristics from the Think Aloud interviews.

| Characteristics | N=7 |
| --- | --- |
| Males/females | 2/5 |
| Mean age (SD) | 58 (± 6.2) |
| Region |  |
| North West | 2 |
| East Midlands | 1 |
| South West | 2 |
| South East | 2 |
| Level of Education |  |
| GCSE, Standard Grade, O-level or equivalent (GNVQ/NVQ Intermediate or Foundation, GSVQ (Level 1 or 2) or RSA Diploma) | 1 |
| A-level, Higher Grade, or equivalent (GNVQ/NVQ Advanced, GSVQ/SVQ (Level 3) or RSA Advanced Diploma) | 1 |
| HNC, HND, SVQ (Level 4 or 5) or RSA Higher Diploma | 1 |
| Postgraduate degree or equivalent | 4 |
| Employment Status |  |
| Employed – Full Time | 3 |
| Unemployed looking for work | 1 |
| Retired | 3 |
| Cancer stage |  |
| IV | 7 |
| Digital Competency |  |
| Not very competent | 1 |
| Somewhat competent | 2 |
| Pretty competent | 2 |
| Highly competent | 2 |
| Engagement with digital technology |  |
| Multiple times per day | 5 |
| At least once per day | 2 |
| Devices used |  |
| Smartphone | 6 |
| Computer/Laptop | 7 |
| Tablet | 4 |
| Smart TV | 5 |
| Smart Watch | 1 |

Note: GCSE, General Certificate in Secondary Education; O-level, Ordinary-level (now known GCSE) GNVQ, General National Vocational Qualification; NVQ, National Vocational Qualification; GSVQ, General Scottish Vocational Qualifications; RSA, Royal Society of Arts; A-level, Advanced-Level SVQ, Scottish Vocational Qualifications; HNC, Higher National Certificate; HND, Higher National Diploma.

Table 2: Illustrating the SUS score and rating given per participant for the use of ExerciseGuide UK.

| Participant | SUS Score | Rating |
| --- | --- | --- |
| 1 | 87.5 | Excellent |
| 2 | 75 | Good |
| 3 | 67.5 | Ok |
| 4 | 17.5 | Awful |
| 5 | 90 | Excellent |
| 6 | 85 | Excellent |
| 7 | 82.5 | Excellent |

Table 3: Demonstrating overall rankings per question for the System Usability Scale following the Think Aloud interviews.

|  | *Strongly Disagree* | *Disagree* | *Neutral* | *Agree* | *Strongly Agree* |
| --- | --- | --- | --- | --- | --- |
| *I think that I would like to use this website frequently* | *0* | *1* | *3* | *1* | *2* |
| *I found this website unnecessarily complex* | *4* | *1* | *1* | *1* | *0* |
| *I thought this website was easy to use* | *0* | *1* | *1* | *4* | *1* |
| *I think that I would need assistance to be able to use this website* | *3* | *2* | *0* | *1* | *1* |
| *I found the various functions in this website were well integrated* | *0* | *2* | *4* | *1* | *0* |
| *I thought there was too much inconsistency in this website* | *5* | *1* | *0* | *1* | *0* |
| *I would imagine that most people would learn to use this website very quickly* | *0* | *1* | *0* | *5* | *1* |
| *I found this website very cumbersome/awkward to use* | *4* | *2* | *0* | *0* | *1* |
| *I felt very confident using this website* | *0* | *1* | *1* | *2* | *3* |
| *I needed to learn a lot of things before I could get going with this website* | *6* | *0* | *0* | *0* | *1* |

1. Sharp H, Rogers Y, Preece J. Interaction Design: Beyond Human-Computer Interaction: Wiley; 2007.
